# Retraction: Association of body weight and physical activity with blood pressure in a rural population in the Dikgale village of Limpopo Province in South Africa

**DOI:** 10.1186/1756-0500-6-422

**Published:** 2013-10-18

**Authors:** Seth S Mkhonto, Demetre Labadarios, Musawenkosi LH Mabaso

**Affiliations:** 1 Population Health, Health Systems and Innovation, Human Sciences Research Council, 134 Pretorius Street, Pretoria 0002, South Africa; 2 Department of Medical Science, University of the Limpopo, Turfloop Campus, Fauna Park, Polokwane 0787, South Africa; 3 HIV/AIDS, STIs and TB, Human Sciences Research Council, 750 Francois Road, Durban 4001, South Africa

## 

This article [1] has been retracted by the Editor because the authors do not have ownership of the data they report. A formal investigation conducted by the University of Limpopo, South Africa, has concluded that the data reported in this article are the sole property of the University of Limpopo.
